# Cs_5_Sn_9_(OH)·4NH_3_

**DOI:** 10.1107/S1600536814011817

**Published:** 2014-05-31

**Authors:** Ute Friedrich, Nikolaus Korber

**Affiliations:** a Institut für Anorganische Chemie, Universität Regensburg, Universitätsstrasse 31, 93053 Regensburg, Germany

## Abstract

The title compound, penta­caesium nona­stannide hydroxide tetra­ammonia, crystallized from a solution of CsSnBi in liquid ammonia. The Sn_9_^4−^ unit forms a monocapped quadratic anti­prism. The hydroxide ion is surrounded by five caesium cations, which form a distorted quadratic pyramidal polyhedron. A three-dimensional network is formed by Cs—Sn [3.8881 (7) Å to 4.5284 (7) Å] and Cs—NH_3_ [3.276 (7)–3.636 (7) Å] contacts.

## Related literature   

For the co-crystallization of Zintl anions and oxide or hydroxide ions see, for example: Boss *et al.* (2005 ▶), Röhr (1995 ▶) For the diagonal ratio value of the Sn_9_^4−^ anion, see: Fässler & Hoffmann (1999 ▶).

## Experimental   

### 

#### Crystal data  

Cs_5_Sn_9_(OH)·4NH_3_
*M* *_r_* = 1817.90
Orthorhombic, 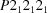

*a* = 10.0935 (1) Å
*b* = 14.8256 (2) Å
*c* = 20.0419 (3) Å
*V* = 2999.11 (7) Å^3^
*Z* = 4
Mo *K*α radiation
μ = 13.34 mm^−1^
*T* = 123 K
0.32 × 0.15 × 0.06 mm

#### Data collection  

Agilent SuperNova (single source at offset, Eos) diffractometer
Absorption correction: multi-scan (*CrysAlis PRO*; Agilent, 2012 ▶) *T*_min_ = 0.263, *T*_max_ = 1.000
34752 measured reflections
5910 independent reflections
5754 reflections with *I* > 2σ(*I*)
*R*_int_ = 0.045

#### Refinement  

*R*[*F* ^2^ > 2σ(*F* ^2^)] = 0.020
*wR*(*F* ^2^) = 0.037
*S* = 1.05
5910 reflections
191 parameters
H-atom parameters constrained
Δρ_max_ = 0.69 e Å^−3^
Δρ_min_ = −0.61 e Å^−3^
Absolute structure: Flack *x* determined using 2458 quotients [(*I* ^+^)−(*I* ^−^)]/[(*I* ^+^)+(*I* ^−^)] (Parsons & Flack, 2004 ▶)
Absolute structure parameter: 0.037 (17)

### 

Data collection: *CrysAlis PRO* (Agilent, 2012 ▶); cell refinement: *CrysAlis PRO*; data reduction: *CrysAlis PRO*; program(s) used to solve structure: *OLEX2*.*SOLVE* (Bourhis *et al.*, 2014 ▶); program(s) used to refine structure: *SHELXL2013* (Sheldrick, 2008 ▶); molecular graphics: *DIAMOND* (Brandenburg & Putz, 2011 ▶); software used to prepare material for publication: *OLEX2* (Dolomanov *et al.*, 2009 ▶).

## Supplementary Material

Crystal structure: contains datablock(s) I. DOI: 10.1107/S1600536814011817/ru2058sup1.cif

Structure factors: contains datablock(s) I. DOI: 10.1107/S1600536814011817/ru2058Isup2.hkl

CCDC reference: 1004528

Additional supporting information:  crystallographic information; 3D view; checkCIF report

## Figures and Tables

**Fig. 1.**
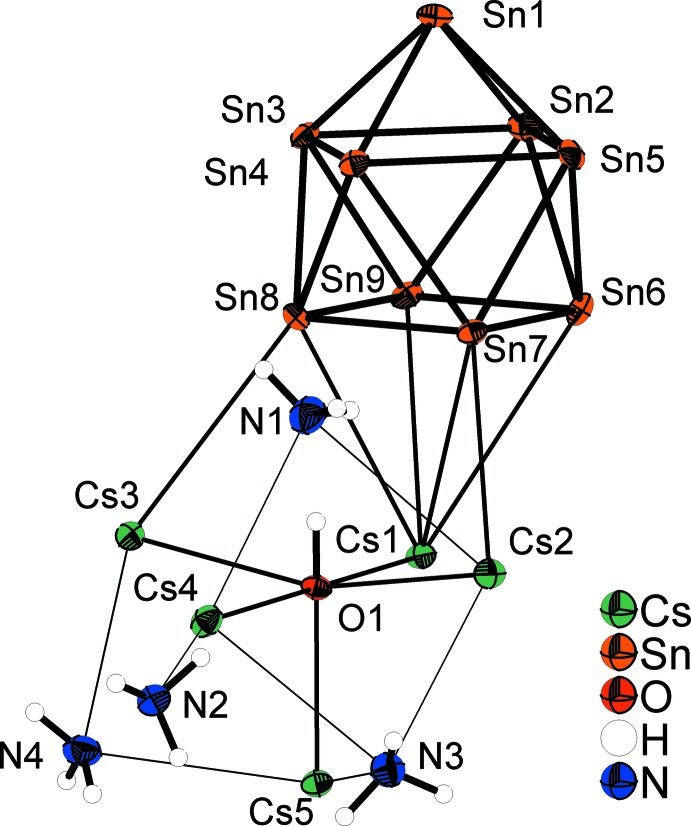
: Asymmetric unit of the title compound; displacement ellipsoids drawn at the 50% probability level.

**Fig. 2.**
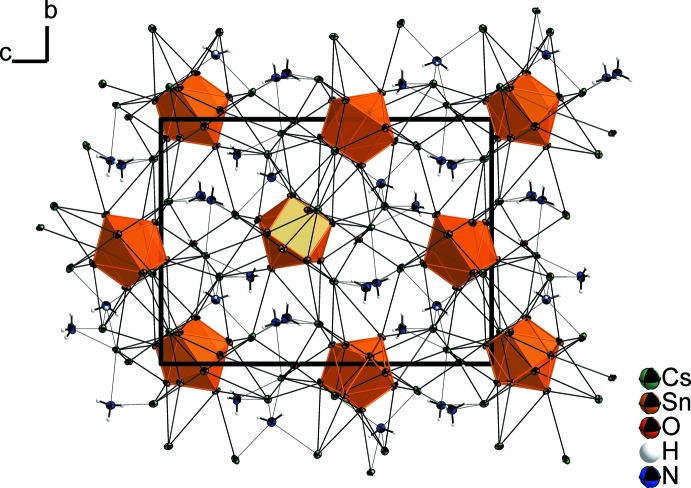
: Projection of the unit cell along the crystallographic a-axis; displacement ellipsoids drawn at the 50% probability level.

## supplementary crystallographic information

### 1. Comment

The crystal structure of Cs_5_Sn_9_(OH) × 4 NH_3_ was determined in the course of solvation experiments on ternary alkali metal–Sn–Bi-phases.

The Sn_9_^4-^ anion has a diagonal ratio value (Fässler *et al.*, 1999) which is very close to 1, consequently it can be described as a monocapped quadratic antiprism (Fig. 1). Sn—Sn bond lengths range from 2.9310 (8) Å to 3.2457 (8) Å. The nonastannid cluster is surrounded by 15 caesium cations with distances ranging from 3.8881 (7) Å to 4.5284 (7) Å. The chemical origin of the hydroxide anion could not be determined, but it is likely that water was introduced to the system, which was deprotonated by the ammonia solvent. The caesium cations form a distorted quadratic pyramide around the hydroxide ion (Fig. 1). The distances *d*_Cs—O_ have values between 2.818 (5) Å and 3.080 (6) Å. Angles between neighbouring equatorial cations Cs_eq_–O(1)–Cs_eq_ range from 86.43 (13)° to 95.28 (17)°, angles between the axial and the equatorial Cs cations Cs_ax_–O(1)–Cs_eq_ have values between 85.15 (15)° and 101.89 (16)°. The ammonia molecules coordinate with distances from 3.276 (7) Å to 3.636 (7) Å to the caesium cations, forming a three dimensional network (Fig. 2).

### 2. Experimental

The educt material CsSnBi was synthesized in glas ampoules at 723 K from stochiometric amounts of the corresponding elements. 0.35 g (0.38 mmol) CsSnBi were weighed in a baked out reaction vessel, afterwards 10 ml of ammonia were condensed, leading to a brownish red solution. The solution was stored for four months at 236 K. The title compound formed as red plate-shaped crystals.

### 3. Refinement

The H atoms of the ammonia molecules and the hydrogen atom of the hydroxide ion were positioned with idealized geometry and refined isotropically with *U*_iso_(H) = 1.2 *U*_eq_(O) and *U*_iso_(H) = 1.5 *U*_eq_(N) using a riding model. For a chemically reasonable alignment of the hydrogen atoms of three ammonia molecules dummy atoms were used. The hydrogen atom of the hydroxide anion was placed in elongation of the Cs(5)–O(1) axes with the HFIX 163 command.

### Crystal data

**Table:**

Cs_5_Sn_9_(OH)·4NH_3_	*D*_x_ = 4.026 Mg m^−^^3^
*M**_r_* = 1817.90	Mo *K*α radiation, λ = 0.71073 Å
Orthorhombic, *P*2_1_2_1_2_1_	Cell parameters from 21366 reflections
*a* = 10.0935 (1) Å	θ = 2.9–28.8°
*b* = 14.8256 (2) Å	µ = 13.34 mm^−^^1^
*c* = 20.0419 (3) Å	*T* = 123 K
*V* = 2999.11 (7) Å^3^	Plate, red
*Z* = 4	0.32 × 0.15 × 0.06 mm
*F*(000) = 3096	

### Data collection

**Table:**

Agilent SuperNova (single source at offset, Eos) diffractometer	5910 independent reflections
Radiation source: SuperNova (Mo) X-ray Source	5754 reflections with *I* > 2σ(*I*)
Detector resolution: 7.9851 pixels mm^-1^	*R*_int_ = 0.045
ω scans	θ_max_ = 26.0°, θ_min_ = 2.9°
Absorption correction: multi-scan (*CrysAlis PRO*; Agilent, 2012)	*h* = −12→12
*T*_min_ = 0.263, *T*_max_ = 1.000	*k* = −18→18
34752 measured reflections	*l* = −24→24

### Refinement

**Table:**

Refinement on *F*^2^	Hydrogen site location: inferred from neighbouring sites
Least-squares matrix: full	H-atom parameters constrained
*R*[*F*^2^ > 2σ(*F*^2^)] = 0.020	*w* = 1/[σ^2^(*F*_o_^2^) + (0.0119*P*)^2^ + 0.8162*P*] where *P* = (*F*_o_^2^ + 2*F*_c_^2^)/3
*wR*(*F*^2^) = 0.037	(Δ/σ)_max_ = 0.002
*S* = 1.05	Δρ_max_ = 0.69 e Å^−^^3^
5910 reflections	Δρ_min_ = −0.61 e Å^−^^3^
191 parameters	Absolute structure: Flack *x* determined using 2458 quotients [(*I*^+^)-(*I*^-^)]/[(*I*^+^)+(*I*^-^)] (Parsons & Flack, 2004)
0 restraints	Absolute structure parameter: 0.037 (17)

### Special details

**Table:**

Experimental. crystal mounting in perfluoretherAbsorption correction: CrysAlisPro (Agilent Technologies, 2012), Empirical absorption correction using spherical harmonics, implemented in SCALE3 ABSPACK scaling algorithm.
Geometry. All e.s.d.'s (except the e.s.d. in the dihedral angle between two l.s. planes) are estimated using the full covariance matrix. The cell e.s.d.'s are taken into account individually in the estimation of e.s.d.'s in distances, angles and torsion angles; correlations between e.s.d.'s in cell parameters are only used when they are defined by crystal symmetry. An approximate (isotropic) treatment of cell e.s.d.'s is used for estimating e.s.d.'s involving l.s. planes.
Refinement. Refinement of *F*^2^ against ALL reflections. The weighted *R*- factor *wR* and goodness of fit *S* are based on *F*^2^, conventional *R*-factors *R* are based on *F*, with *F* set to zero for negative *F*^2^. The threshold expression of *F*^2^ > σ(*F*^2^) is used only for calculating *R*-factors(gt) *etc*. and is not relevant to the choice of reflections for refinement. *R*-factors based on *F*^2^ are statistically about twice as large as those based on *F*, and *R*-factors based on ALL data will be even larger.

### Fractional atomic coordinates and isotropic or equivalent isotropic displacement parameters (Å^2^)

**Table:**

	*x*	*y*	*z*	*U*_iso_*/*U*_eq_	
Cs1	0.69651 (4)	0.62993 (3)	0.52616 (3)	0.01745 (12)	
Cs5	0.96022 (5)	0.55715 (3)	0.36506 (3)	0.01733 (12)	
Cs2	0.77685 (5)	0.33699 (3)	0.47723 (3)	0.01964 (13)	
Cs3	0.53343 (5)	0.64420 (3)	0.32331 (3)	0.01897 (12)	
Cs4	0.70495 (5)	0.39111 (4)	0.27494 (3)	0.02169 (13)	
Sn8	0.32179 (5)	0.57700 (4)	0.48243 (3)	0.01753 (13)	
Sn9	0.33484 (5)	0.68860 (4)	0.60507 (3)	0.01726 (14)	
Sn6	0.44704 (5)	0.53549 (4)	0.67889 (3)	0.01920 (14)	
Sn4	0.15595 (5)	0.42149 (4)	0.51683 (3)	0.01634 (13)	
Sn5	0.24692 (5)	0.38957 (4)	0.66732 (3)	0.01944 (14)	
Sn3	0.07763 (5)	0.62769 (4)	0.55430 (3)	0.01715 (14)	
Sn2	0.17196 (6)	0.59083 (4)	0.70575 (3)	0.02052 (14)	
Sn7	0.43672 (5)	0.42314 (4)	0.55797 (3)	0.01669 (13)	
Sn1	−0.00956 (5)	0.47064 (4)	0.63144 (3)	0.01970 (14)	
O1	0.7008 (5)	0.5080 (3)	0.4020 (3)	0.0160 (13)	
H1	0.6133	0.4914	0.4145	0.019*	
N4	0.8001 (7)	0.6444 (5)	0.2302 (4)	0.0272 (19)	
H4A	0.8659	0.6084	0.2143	0.041*	
H4B	0.8275	0.7029	0.2296	0.041*	
H4C	0.7269	0.6384	0.2041	0.041*	
N3	0.9939 (7)	0.3306 (4)	0.3478 (4)	0.0249 (19)	
H3A	1.0352	0.3496	0.3100	0.037*	
H3B	0.9629	0.2735	0.3416	0.037*	
H3C	1.0524	0.3310	0.3823	0.037*	
N2	0.7994 (6)	0.2374 (4)	0.1655 (4)	0.0237 (18)	
H2A	0.7623	0.2044	0.1321	0.036*	
H2B	0.7658	0.2189	0.2054	0.036*	
H2C	0.8888	0.2293	0.1653	0.036*	
N1	0.4815 (7)	0.3126 (5)	0.3796 (4)	0.031 (2)	
H1A	0.4909	0.2518	0.3834	0.046*	
H1B	0.3990	0.3256	0.3640	0.046*	
H1C	0.4929	0.3388	0.4202	0.046*	

### Atomic displacement parameters (Å^2^)

**Table:**

	*U*^11^	*U*^22^	*U*^33^	*U*^12^	*U*^13^	*U*^23^
Cs1	0.0134 (2)	0.0200 (3)	0.0189 (3)	−0.0008 (2)	0.0009 (2)	−0.0020 (2)
Cs5	0.0155 (2)	0.0163 (3)	0.0202 (3)	−0.0019 (2)	−0.0001 (2)	−0.0021 (2)
Cs2	0.0186 (3)	0.0152 (2)	0.0251 (4)	0.0012 (2)	0.0014 (2)	0.0016 (2)
Cs3	0.0175 (2)	0.0210 (3)	0.0185 (3)	0.0007 (2)	−0.0004 (2)	0.0003 (2)
Cs4	0.0192 (3)	0.0264 (3)	0.0194 (3)	0.0021 (2)	−0.0026 (2)	−0.0053 (2)
Sn8	0.0175 (3)	0.0204 (3)	0.0146 (4)	−0.0020 (2)	0.0026 (3)	0.0044 (3)
Sn9	0.0146 (3)	0.0119 (3)	0.0253 (4)	−0.0024 (2)	−0.0030 (3)	−0.0010 (3)
Sn6	0.0169 (3)	0.0215 (3)	0.0192 (4)	0.0014 (2)	−0.0077 (3)	−0.0012 (3)
Sn4	0.0154 (3)	0.0157 (3)	0.0179 (4)	−0.0031 (2)	−0.0020 (3)	−0.0035 (3)
Sn5	0.0221 (3)	0.0161 (3)	0.0201 (4)	0.0003 (2)	0.0027 (3)	0.0078 (3)
Sn3	0.0114 (3)	0.0154 (3)	0.0247 (4)	0.0013 (2)	−0.0036 (3)	0.0026 (3)
Sn2	0.0195 (3)	0.0238 (3)	0.0183 (4)	−0.0012 (2)	0.0058 (3)	−0.0072 (3)
Sn7	0.0129 (3)	0.0178 (3)	0.0194 (4)	0.0031 (2)	0.0017 (2)	−0.0013 (3)
Sn1	0.0141 (3)	0.0207 (3)	0.0243 (4)	−0.0038 (2)	0.0054 (3)	0.0000 (3)
O1	0.015 (3)	0.014 (3)	0.019 (4)	−0.004 (2)	0.001 (2)	0.000 (3)
N4	0.023 (4)	0.033 (4)	0.026 (5)	0.000 (3)	0.006 (4)	−0.004 (4)
N3	0.026 (4)	0.021 (4)	0.027 (5)	−0.001 (3)	0.000 (4)	0.001 (4)
N2	0.020 (4)	0.026 (4)	0.025 (5)	0.002 (3)	0.000 (3)	0.001 (4)
N1	0.029 (4)	0.030 (4)	0.034 (6)	−0.007 (4)	−0.007 (4)	0.001 (4)

### Geometric parameters (Å, º)

**Table:**

Cs1—N2^i^	3.417 (8)	Sn9—Sn6	2.9367 (8)
Cs1—Sn3^ii^	3.8881 (7)	Sn9—Sn2	2.9793 (9)
Cs1—Sn8	3.9609 (7)	Sn9—Cs1^xii^	4.0131 (8)
Cs1—Sn9^iii^	4.0132 (8)	Sn9—Cs5^xii^	4.0210 (7)
Cs1—Sn9	4.0723 (7)	Sn9—Cs3^xii^	4.1787 (8)
Cs1—Sn7	4.0843 (8)	Sn6—Sn7	2.9425 (9)
Cs1—Sn3^iii^	4.1175 (8)	Sn6—Sn2	2.9449 (8)
Cs1—Sn6	4.2037 (9)	Sn6—Sn5	2.9688 (8)
Cs1—Cs5	4.3213 (8)	Sn6—Cs5^i^	4.0847 (9)
Cs1—Sn1^ii^	4.3395 (8)	Sn6—Cs4^i^	4.1507 (8)
Cs5—O1	2.817 (5)	Sn4—Sn1	2.9323 (9)
Cs5—N2^iv^	3.661 (6)	Sn4—Sn7	2.9516 (7)
Cs5—Cs4	3.9951 (7)	Sn4—Sn5	3.1880 (9)
Cs5—Sn9^iii^	4.0210 (7)	Sn4—Sn3	3.2457 (8)
Cs5—Sn6^v^	4.0847 (9)	Sn4—Cs2^vii^	4.0234 (7)
Cs5—Sn3^ii^	4.1089 (9)	Sn4—Cs2^xi^	4.1038 (7)
Cs5—Sn4^ii^	4.1474 (8)	Sn4—Cs5^xi^	4.1473 (8)
Cs5—Sn8^ii^	4.3519 (8)	Sn5—Sn1	2.9434 (8)
Cs5—Cs2	4.3741 (7)	Sn5—Sn7	2.9530 (9)
Cs2—Sn7	4.0045 (7)	Sn5—Sn2	3.1732 (8)
Cs2—Sn4^vi^	4.0235 (7)	Sn5—Cs3^xiii^	4.2463 (8)
Cs2—Sn4^ii^	4.1038 (7)	Sn5—Cs4^vii^	4.3400 (8)
Cs2—Cs4	4.1961 (9)	Sn5—Cs2^vii^	4.4459 (8)
Cs2—Sn7^vi^	4.2398 (7)	Sn3—Sn1	2.9301 (8)
Cs2—Sn1^ii^	4.2575 (9)	Sn3—Sn2	3.2277 (9)
Cs2—Sn5^vi^	4.4460 (8)	Sn3—Cs1^xi^	3.8882 (7)
Cs2—Cs2^vii^	5.7408 (5)	Sn3—Cs5^xi^	4.1088 (9)
Cs2—Cs2^vi^	5.7408 (5)	Sn3—Cs1^xii^	4.1174 (8)
Cs3—N2^viii^	3.639 (7)	Sn3—Cs3^xii^	4.2017 (8)
Cs3—Sn8	3.9656 (8)	Sn2—Sn1	2.9581 (9)
Cs3—Sn9^iii^	4.1786 (8)	Sn2—Cs4^xiii^	4.0580 (8)
Cs3—Sn3^iii^	4.2016 (8)	Sn2—Cs3^xii^	4.2103 (7)
Cs3—Sn2^iii^	4.2102 (7)	Sn7—Cs2^vii^	4.2398 (7)
Cs3—Sn1^ix^	4.2123 (9)	Sn1—Cs4^xiii^	4.0449 (9)
Cs3—Cs4	4.2445 (7)	Sn1—Cs3^xiii^	4.2122 (9)
Cs3—Sn5^ix^	4.2463 (8)	Sn1—Cs2^xi^	4.2576 (9)
Cs3—Cs4^viii^	4.8028 (7)	Sn1—Cs1^xi^	4.3396 (8)
Cs4—N1	3.292 (8)	O1—H1	0.9500
Cs4—Sn1^ix^	4.0449 (9)	N4—H4A	0.9100
Cs4—Sn2^ix^	4.0579 (8)	N4—H4B	0.9100
Cs4—Sn6^v^	4.1507 (8)	N4—H4C	0.9100
Cs4—Sn5^vi^	4.3399 (8)	N3—H3A	0.9100
Cs4—Cs3^x^	4.8027 (7)	N3—H3B	0.9100
Sn8—Sn4	2.9314 (8)	N3—H3C	0.9100
Sn8—Sn3	2.9518 (8)	N2—H2A	0.9100
Sn8—Sn9	2.9658 (9)	N2—H2B	0.9100
Sn8—Sn7	2.9735 (8)	N2—H2C	0.9100
Sn8—Cs5^xi^	4.3518 (8)	N1—H1A	0.9100
Sn8—Cs1^xii^	4.5284 (7)	N1—H1B	0.9100
Sn9—Sn3	2.9310 (8)	N1—H1C	0.9100
			
N2^i^—Cs1—Sn3^ii^	82.78 (11)	Sn4—Sn8—Cs3	133.97 (2)
N2^i^—Cs1—Sn8	107.85 (11)	Sn3—Sn8—Cs3	141.10 (2)
Sn3^ii^—Cs1—Sn8	167.198 (19)	Sn9—Sn8—Cs3	120.16 (2)
N2^i^—Cs1—Sn9^iii^	98.37 (12)	Sn7—Sn8—Cs3	113.08 (2)
Sn3^ii^—Cs1—Sn9^iii^	75.913 (15)	Cs1—Sn8—Cs3	67.283 (14)
Sn8—Cs1—Sn9^iii^	108.664 (18)	Sn4—Sn8—Cs5^xi^	66.113 (17)
N2^i^—Cs1—Sn9	64.56 (11)	Sn3—Sn8—Cs5^xi^	65.218 (18)
Sn3^ii^—Cs1—Sn9	146.35 (2)	Sn9—Sn8—Cs5^xi^	121.53 (2)
Sn8—Cs1—Sn9	43.304 (13)	Sn7—Sn8—Cs5^xi^	123.40 (2)
Sn9^iii^—Cs1—Sn9	115.038 (15)	Cs1—Sn8—Cs5^xi^	159.03 (2)
N2^i^—Cs1—Sn7	108.20 (12)	Cs3—Sn8—Cs5^xi^	91.952 (17)
Sn3^ii^—Cs1—Sn7	127.309 (17)	Sn4—Sn8—Cs1^xii^	127.17 (2)
Sn8—Cs1—Sn7	43.347 (12)	Sn3—Sn8—Cs1^xii^	62.688 (16)
Sn9^iii^—Cs1—Sn7	145.99 (2)	Sn9—Sn8—Cs1^xii^	60.572 (16)
Sn9—Cs1—Sn7	61.588 (12)	Sn7—Sn8—Cs1^xii^	149.81 (2)
N2^i^—Cs1—Sn3^iii^	79.70 (12)	Cs1—Sn8—Cs1^xii^	94.875 (13)
Sn3^ii^—Cs1—Sn3^iii^	110.648 (15)	Cs3—Sn8—Cs1^xii^	83.040 (14)
Sn8—Cs1—Sn3^iii^	78.928 (14)	Cs5^xi^—Sn8—Cs1^xii^	79.056 (13)
Sn9^iii^—Cs1—Sn3^iii^	42.236 (12)	Sn3—Sn9—Sn6	106.16 (2)
Sn9—Cs1—Sn3^iii^	72.811 (14)	Sn3—Sn9—Sn8	60.07 (2)
Sn7—Cs1—Sn3^iii^	121.949 (15)	Sn6—Sn9—Sn8	90.20 (2)
N2^i^—Cs1—Sn6	66.66 (12)	Sn3—Sn9—Sn2	66.20 (2)
Sn3^ii^—Cs1—Sn6	118.956 (19)	Sn6—Sn9—Sn2	59.70 (2)
Sn8—Cs1—Sn6	61.522 (14)	Sn8—Sn9—Sn2	105.39 (2)
Sn9^iii^—Cs1—Sn6	155.360 (17)	Sn3—Sn9—Cs1^xii^	70.784 (18)
Sn9—Cs1—Sn6	41.531 (12)	Sn6—Sn9—Cs1^xii^	169.28 (3)
Sn7—Cs1—Sn6	41.560 (13)	Sn8—Sn9—Cs1^xii^	79.36 (2)
Sn3^iii^—Cs1—Sn6	113.663 (16)	Sn2—Sn9—Cs1^xii^	125.31 (2)
N2^i^—Cs1—Cs5	138.34 (11)	Sn3—Sn9—Cs5^xii^	128.26 (2)
Sn3^ii^—Cs1—Cs5	59.790 (14)	Sn6—Sn9—Cs5^xii^	121.89 (2)
Sn8—Cs1—Cs5	111.923 (17)	Sn8—Sn9—Cs5^xii^	131.32 (2)
Sn9^iii^—Cs1—Cs5	57.548 (12)	Sn2—Sn9—Cs5^xii^	121.93 (2)
Sn9—Cs1—Cs5	153.592 (18)	Cs1^xii^—Sn9—Cs5^xii^	65.078 (14)
Sn7—Cs1—Cs5	108.945 (16)	Sn3—Sn9—Cs1	126.40 (2)
Sn3^iii^—Cs1—Cs5	96.014 (16)	Sn6—Sn9—Cs1	71.633 (18)
Sn6—Cs1—Cs5	146.084 (16)	Sn8—Sn9—Cs1	66.348 (17)
N2^i^—Cs1—Sn1^ii^	84.70 (11)	Sn2—Sn9—Cs1	130.83 (2)
Sn3^ii^—Cs1—Sn1^ii^	41.268 (12)	Cs1^xii^—Sn9—Cs1	101.571 (16)
Sn8—Cs1—Sn1^ii^	130.739 (17)	Cs5^xii^—Sn9—Cs1	88.619 (15)
Sn9^iii^—Cs1—Sn1^ii^	116.464 (15)	Sn3—Sn9—Cs3^xii^	69.949 (18)
Sn9—Cs1—Sn1^ii^	122.702 (19)	Sn6—Sn9—Cs3^xii^	124.49 (3)
Sn7—Cs1—Sn1^ii^	87.394 (15)	Sn8—Sn9—Cs3^xii^	125.68 (2)
Sn3^iii^—Cs1—Sn1^ii^	149.840 (16)	Sn2—Sn9—Cs3^xii^	69.766 (18)
Sn6—Cs1—Sn1^ii^	82.772 (15)	Cs1^xii^—Sn9—Cs3^xii^	64.795 (14)
Cs5—Cs1—Sn1^ii^	78.830 (14)	Cs5^xii^—Sn9—Cs3^xii^	67.783 (13)
O1—Cs5—N2^iv^	148.09 (15)	Cs1—Sn9—Cs3^xii^	155.890 (17)
O1—Cs5—Cs4	50.21 (11)	Sn9—Sn6—Sn7	90.51 (2)
N2^iv^—Cs5—Cs4	143.27 (13)	Sn9—Sn6—Sn2	60.87 (2)
O1—Cs5—Sn9^iii^	84.88 (10)	Sn7—Sn6—Sn2	105.96 (2)
N2^iv^—Cs5—Sn9^iii^	63.22 (10)	Sn9—Sn6—Sn5	105.16 (2)
Cs4—Cs5—Sn9^iii^	116.228 (16)	Sn7—Sn6—Sn5	59.94 (2)
O1—Cs5—Sn6^v^	111.48 (12)	Sn2—Sn6—Sn5	64.902 (19)
N2^iv^—Cs5—Sn6^v^	86.59 (13)	Sn9—Sn6—Cs5^i^	143.95 (3)
Cs4—Cs5—Sn6^v^	61.811 (14)	Sn7—Sn6—Cs5^i^	124.76 (2)
Sn9^iii^—Cs5—Sn6^v^	121.556 (19)	Sn2—Sn6—Cs5^i^	98.21 (2)
O1—Cs5—Sn3^ii^	95.23 (12)	Sn5—Sn6—Cs5^i^	88.99 (2)
N2^iv^—Cs5—Sn3^ii^	77.16 (13)	Sn9—Sn6—Cs4^i^	110.94 (2)
Cs4—Cs5—Sn3^ii^	139.478 (18)	Sn7—Sn6—Cs4^i^	124.08 (2)
Sn9^iii^—Cs5—Sn3^ii^	73.429 (15)	Sn2—Sn6—Cs4^i^	129.80 (2)
Sn6^v^—Cs5—Sn3^ii^	149.616 (17)	Sn5—Sn6—Cs4^i^	143.42 (2)
O1—Cs5—Sn4^ii^	97.15 (11)	Cs5^i^—Sn6—Cs4^i^	58.034 (13)
N2^iv^—Cs5—Sn4^ii^	99.27 (12)	Sn9—Sn6—Cs1	66.836 (19)
Cs4—Cs5—Sn4^ii^	109.876 (16)	Sn7—Sn6—Cs1	67.046 (18)
Sn9^iii^—Cs5—Sn4^ii^	119.695 (19)	Sn2—Sn6—Cs1	127.23 (2)
Sn6^v^—Cs5—Sn4^ii^	113.434 (16)	Sn5—Sn6—Cs1	126.39 (2)
Sn3^ii^—Cs5—Sn4^ii^	46.295 (12)	Cs5^i^—Sn6—Cs1	129.785 (16)
O1—Cs5—Cs1	45.22 (11)	Cs4^i^—Sn6—Cs1	75.135 (14)
N2^iv^—Cs5—Cs1	110.56 (12)	Sn8—Sn4—Sn1	108.31 (2)
Cs4—Cs5—Cs1	95.417 (14)	Sn8—Sn4—Sn7	60.719 (19)
Sn9^iii^—Cs5—Cs1	57.374 (13)	Sn1—Sn4—Sn7	109.03 (3)
Sn6^v^—Cs5—Cs1	155.179 (17)	Sn8—Sn4—Sn5	100.07 (2)
Sn3^ii^—Cs5—Cs1	54.861 (13)	Sn1—Sn4—Sn5	57.31 (2)
Sn4^ii^—Cs5—Cs1	82.331 (15)	Sn7—Sn4—Sn5	57.344 (19)
O1—Cs5—Sn8^ii^	130.91 (12)	Sn8—Sn4—Sn3	56.818 (17)
N2^iv^—Cs5—Sn8^ii^	59.02 (12)	Sn1—Sn4—Sn3	56.350 (19)
Cs4—Cs5—Sn8^ii^	145.780 (16)	Sn7—Sn4—Sn3	99.29 (2)
Sn9^iii^—Cs5—Sn8^ii^	96.897 (15)	Sn5—Sn4—Sn3	89.49 (2)
Sn6^v^—Cs5—Sn8^ii^	108.922 (16)	Sn8—Sn4—Cs2^vii^	125.66 (2)
Sn3^ii^—Cs5—Sn8^ii^	40.711 (12)	Sn1—Sn4—Cs2^vii^	112.72 (2)
Sn4^ii^—Cs5—Sn8^ii^	40.262 (11)	Sn7—Sn4—Cs2^vii^	73.050 (17)
Cs1—Cs5—Sn8^ii^	95.499 (16)	Sn5—Sn4—Cs2^vii^	75.125 (17)
O1—Cs5—Cs2	43.82 (10)	Sn3—Sn4—Cs2^vii^	164.60 (3)
N2^iv^—Cs5—Cs2	155.66 (13)	Sn8—Sn4—Cs2^xi^	136.64 (2)
Cs4—Cs5—Cs2	59.978 (14)	Sn1—Sn4—Cs2^xi^	72.311 (18)
Sn9^iii^—Cs5—Cs2	119.331 (17)	Sn7—Sn4—Cs2^xi^	162.15 (2)
Sn6^v^—Cs5—Cs2	108.391 (15)	Sn5—Sn4—Cs2^xi^	113.97 (2)
Sn3^ii^—Cs5—Cs2	80.651 (15)	Sn3—Sn4—Cs2^xi^	96.036 (17)
Sn4^ii^—Cs5—Cs2	57.504 (12)	Cs2^vii^—Sn4—Cs2^xi^	89.873 (12)
Cs1—Cs5—Cs2	62.723 (12)	Sn8—Sn4—Cs5^xi^	73.625 (18)
Sn8^ii^—Cs5—Cs2	97.327 (15)	Sn1—Sn4—Cs5^xi^	100.52 (2)
Sn7—Cs2—Sn4^vi^	91.823 (15)	Sn7—Sn4—Cs5^xi^	131.17 (2)
Sn7—Cs2—Sn4^ii^	128.598 (19)	Sn5—Sn4—Cs5^xi^	154.56 (2)
Sn4^vi^—Cs2—Sn4^ii^	124.596 (16)	Sn3—Sn4—Cs5^xi^	66.228 (17)
Sn7—Cs2—Cs4	100.440 (16)	Cs2^vii^—Sn4—Cs5^xi^	128.909 (19)
Sn4^vi^—Cs2—Cs4	99.106 (17)	Cs2^xi^—Sn4—Cs5^xi^	64.025 (13)
Sn4^ii^—Cs2—Cs4	106.844 (17)	Sn1—Sn5—Sn7	108.69 (3)
Sn7—Cs2—Sn7^vi^	133.131 (16)	Sn1—Sn5—Sn6	108.66 (2)
Sn4^vi^—Cs2—Sn7^vi^	41.753 (11)	Sn7—Sn5—Sn6	59.59 (2)
Sn4^ii^—Cs2—Sn7^vi^	87.418 (14)	Sn1—Sn5—Sn2	57.697 (19)
Cs4—Cs2—Sn7^vi^	94.530 (17)	Sn7—Sn5—Sn2	100.16 (2)
Sn7—Cs2—Sn1^ii^	89.560 (17)	Sn6—Sn5—Sn2	57.184 (18)
Sn4^vi^—Cs2—Sn1^ii^	125.124 (19)	Sn1—Sn5—Sn4	56.97 (2)
Sn4^ii^—Cs2—Sn1^ii^	41.007 (13)	Sn7—Sn5—Sn4	57.300 (19)
Cs4—Cs2—Sn1^ii^	134.426 (17)	Sn6—Sn5—Sn4	99.31 (2)
Sn7^vi^—Cs2—Sn1^ii^	110.573 (16)	Sn2—Sn5—Sn4	91.23 (2)
Sn7—Cs2—Cs5	109.417 (15)	Sn1—Sn5—Cs3^xiii^	69.015 (19)
Sn4^vi^—Cs2—Cs5	148.679 (19)	Sn7—Sn5—Cs3^xiii^	176.91 (2)
Sn4^ii^—Cs2—Cs5	58.471 (13)	Sn6—Sn5—Cs3^xiii^	118.80 (2)
Cs4—Cs2—Cs5	55.525 (13)	Sn2—Sn5—Cs3^xiii^	76.893 (18)
Sn7^vi^—Cs2—Cs5	115.608 (16)	Sn4—Sn5—Cs3^xiii^	121.47 (2)
Sn1^ii^—Cs2—Cs5	79.136 (14)	Sn1—Sn5—Cs4^vii^	111.766 (19)
Sn7—Cs2—Sn5^vi^	116.490 (16)	Sn7—Sn5—Cs4^vii^	115.01 (2)
Sn4^vi^—Cs2—Sn5^vi^	43.871 (13)	Sn6—Sn5—Cs4^vii^	138.10 (2)
Sn4^ii^—Cs2—Sn5^vi^	114.834 (16)	Sn2—Sn5—Cs4^vii^	144.45 (2)
Cs4—Cs2—Sn5^vi^	60.207 (13)	Sn4—Sn5—Cs4^vii^	111.50 (2)
Sn7^vi^—Cs2—Sn5^vi^	39.663 (12)	Cs3^xiii^—Sn5—Cs4^vii^	68.012 (13)
Sn1^ii^—Cs2—Sn5^vi^	149.209 (16)	Sn1—Sn5—Cs2^vii^	102.07 (2)
Cs5—Cs2—Sn5^vi^	104.930 (17)	Sn7—Sn5—Cs2^vii^	66.404 (18)
Sn7—Cs2—Cs1	56.821 (12)	Sn6—Sn5—Cs2^vii^	123.73 (2)
Sn4^vi^—Cs2—Cs1	148.568 (16)	Sn2—Sn5—Cs2^vii^	152.23 (2)
Sn4^ii^—Cs2—Cs1	80.342 (13)	Sn4—Sn5—Cs2^vii^	61.004 (16)
Cs4—Cs2—Cs1	89.708 (14)	Cs3^xiii^—Sn5—Cs2^vii^	115.845 (16)
Sn7^vi^—Cs2—Cs1	167.741 (16)	Cs4^vii^—Sn5—Cs2^vii^	57.042 (13)
Sn1^ii^—Cs2—Cs1	59.123 (12)	Sn1—Sn3—Sn9	109.13 (3)
Cs5—Cs2—Cs1	58.068 (11)	Sn1—Sn3—Sn8	107.82 (2)
Sn5^vi^—Cs2—Cs1	148.656 (18)	Sn9—Sn3—Sn8	60.55 (2)
Sn7—Cs2—Cs2^vii^	47.576 (10)	Sn1—Sn3—Sn2	57.17 (2)
Sn4^vi^—Cs2—Cs2^vii^	45.631 (12)	Sn9—Sn3—Sn2	57.62 (2)
Sn4^ii^—Cs2—Cs2^vii^	157.83 (2)	Sn8—Sn3—Sn2	99.76 (2)
Cs4—Cs2—Cs2^vii^	95.020 (15)	Sn1—Sn3—Sn4	56.415 (19)
Sn7^vi^—Cs2—Cs2^vii^	87.268 (15)	Sn9—Sn3—Sn4	98.90 (2)
Sn1^ii^—Cs2—Cs2^vii^	122.60 (2)	Sn8—Sn3—Sn4	56.218 (17)
Cs5—Cs2—Cs2^vii^	142.091 (16)	Sn2—Sn3—Sn4	89.22 (2)
Sn5^vi^—Cs2—Cs2^vii^	72.807 (14)	Sn1—Sn3—Cs1^xi^	77.654 (18)
Cs1—Cs2—Cs2^vii^	103.840 (12)	Sn9—Sn3—Cs1^xi^	157.53 (2)
Sn7—Cs2—Cs2^vi^	146.38 (2)	Sn8—Sn3—Cs1^xi^	139.24 (3)
Sn4^vi^—Cs2—Cs2^vi^	80.443 (14)	Sn2—Sn3—Cs1^xi^	115.45 (2)
Sn4^ii^—Cs2—Cs2^vi^	44.495 (8)	Sn4—Sn3—Cs1^xi^	102.444 (18)
Cs4—Cs2—Cs2^vi^	113.053 (17)	Sn1—Sn3—Cs5^xi^	101.43 (2)
Sn7^vi^—Cs2—Cs2^vi^	44.205 (11)	Sn9—Sn3—Cs5^xi^	130.87 (2)
Sn1^ii^—Cs2—Cs2^vi^	69.422 (13)	Sn8—Sn3—Cs5^xi^	74.07 (2)
Cs5—Cs2—Cs2^vi^	92.580 (11)	Sn2—Sn3—Cs5^xi^	155.51 (2)
Sn5^vi^—Cs2—Cs2^vi^	79.856 (14)	Sn4—Sn3—Cs5^xi^	67.477 (17)
Cs1—Cs2—Cs2^vi^	123.659 (15)	Cs1^xi^—Sn3—Cs5^xi^	65.349 (14)
Cs2^vii^—Cs2—Cs2^vi^	123.070 (17)	Sn1—Sn3—Cs1^xii^	170.99 (3)
N2^viii^—Cs3—Sn8	63.17 (13)	Sn9—Sn3—Cs1^xii^	66.980 (17)
N2^viii^—Cs3—Sn9^iii^	115.20 (10)	Sn8—Sn3—Cs1^xii^	77.744 (18)
Sn8—Cs3—Sn9^iii^	105.364 (18)	Sn2—Sn3—Cs1^xii^	115.47 (2)
N2^viii^—Cs3—Sn3^iii^	75.92 (11)	Sn4—Sn3—Cs1^xii^	131.33 (2)
Sn8—Cs3—Sn3^iii^	77.873 (16)	Cs1^xi^—Sn3—Cs1^xii^	102.944 (15)
Sn9^iii^—Cs3—Sn3^iii^	40.943 (12)	Cs5^xi^—Sn3—Cs1^xii^	86.825 (17)
N2^viii^—Cs3—Sn2^iii^	87.77 (10)	Sn1—Sn3—Cs3^xii^	107.44 (2)
Sn8—Cs3—Sn2^iii^	121.638 (18)	Sn9—Sn3—Cs3^xii^	69.109 (18)
Sn9^iii^—Cs3—Sn2^iii^	41.602 (12)	Sn8—Sn3—Cs3^xii^	125.34 (2)
Sn3^iii^—Cs3—Sn2^iii^	45.128 (14)	Sn2—Sn3—Cs3^xii^	67.578 (17)
N2^viii^—Cs3—Sn1^ix^	99.02 (13)	Sn4—Sn3—Cs3^xii^	156.79 (2)
Sn8—Cs3—Sn1^ix^	126.998 (17)	Cs1^xi^—Sn3—Cs3^xii^	88.437 (15)
Sn9^iii^—Cs3—Sn1^ix^	126.514 (18)	Cs5^xi^—Sn3—Cs3^xii^	135.489 (17)
Sn3^iii^—Cs3—Sn1^ix^	149.810 (19)	Cs1^xii^—Sn3—Cs3^xii^	63.713 (14)
Sn2^iii^—Cs3—Sn1^ix^	105.654 (18)	Sn6—Sn2—Sn1	108.91 (2)
N2^viii^—Cs3—Cs4	136.56 (10)	Sn6—Sn2—Sn9	59.43 (2)
Sn8—Cs3—Cs4	100.445 (16)	Sn1—Sn2—Sn9	107.09 (3)
Sn9^iii^—Cs3—Cs4	107.813 (15)	Sn6—Sn2—Sn5	57.914 (18)
Sn3^iii^—Cs3—Cs4	143.281 (17)	Sn1—Sn2—Sn5	57.249 (18)
Sn2^iii^—Cs3—Cs4	131.129 (17)	Sn9—Sn2—Sn5	99.29 (2)
Sn1^ix^—Cs3—Cs4	57.148 (13)	Sn6—Sn2—Sn3	98.82 (2)
N2^viii^—Cs3—Sn5^ix^	58.32 (13)	Sn1—Sn2—Sn3	56.34 (2)
Sn8—Cs3—Sn5^ix^	101.741 (16)	Sn9—Sn2—Sn3	56.183 (19)
Sn9^iii^—Cs3—Sn5^ix^	143.904 (18)	Sn5—Sn2—Sn3	90.07 (2)
Sn3^iii^—Cs3—Sn5^ix^	126.547 (16)	Sn6—Sn2—Cs4^xiii^	164.74 (3)
Sn2^iii^—Cs3—Sn5^ix^	103.266 (17)	Sn1—Sn2—Cs4^xiii^	68.353 (18)
Sn1^ix^—Cs3—Sn5^ix^	40.724 (12)	Sn9—Sn2—Cs4^xiii^	135.78 (2)
Cs4—Cs3—Sn5^ix^	89.967 (15)	Sn5—Sn2—Cs4^xiii^	111.62 (2)
N2^viii^—Cs3—Cs1	107.90 (13)	Sn3—Sn2—Cs4^xiii^	91.928 (19)
Sn8—Cs3—Cs1	56.307 (13)	Sn6—Sn2—Cs3^xii^	123.22 (2)
Sn9^iii^—Cs3—Cs1	55.779 (13)	Sn1—Sn2—Cs3^xii^	106.66 (2)
Sn3^iii^—Cs3—Cs1	57.211 (13)	Sn9—Sn2—Cs3^xii^	68.632 (17)
Sn2^iii^—Cs3—Cs1	92.782 (15)	Sn5—Sn2—Cs3^xii^	157.36 (3)
Sn1^ix^—Cs3—Cs1	147.903 (16)	Sn3—Sn2—Cs3^xii^	67.296 (17)
Cs4—Cs3—Cs1	90.915 (15)	Cs4^xiii^—Sn2—Cs3^xii^	70.996 (14)
Sn5^ix^—Cs3—Cs1	157.780 (18)	Sn6—Sn7—Sn4	105.59 (2)
N2^viii^—Cs3—Cs5	164.84 (13)	Sn6—Sn7—Sn5	60.47 (2)
Sn8—Cs3—Cs5	106.815 (17)	Sn4—Sn7—Sn5	65.36 (2)
Sn9^iii^—Cs3—Cs5	54.469 (11)	Sn6—Sn7—Sn8	89.94 (2)
Sn3^iii^—Cs3—Cs5	91.162 (14)	Sn4—Sn7—Sn8	59.306 (18)
Sn2^iii^—Cs3—Cs5	88.612 (13)	Sn5—Sn7—Sn8	104.72 (2)
Sn1^ix^—Cs3—Cs5	96.134 (15)	Sn6—Sn7—Cs2	118.88 (2)
Cs4—Cs3—Cs5	53.719 (11)	Sn4—Sn7—Cs2	135.04 (2)
Sn5^ix^—Cs3—Cs5	136.804 (17)	Sn5—Sn7—Cs2	143.35 (2)
Cs1—Cs3—Cs5	57.588 (12)	Sn8—Sn7—Cs2	111.92 (2)
N2^viii^—Cs3—Cs4^viii^	43.41 (12)	Sn6—Sn7—Cs1	71.394 (18)
Sn8—Cs3—Cs4^viii^	104.556 (15)	Sn4—Sn7—Cs1	125.38 (2)
Sn9^iii^—Cs3—Cs4^viii^	93.062 (13)	Sn5—Sn7—Cs1	131.21 (2)
Sn3^iii^—Cs3—Cs4^viii^	71.279 (13)	Sn8—Sn7—Cs1	66.117 (17)
Sn2^iii^—Cs3—Cs4^viii^	53.023 (12)	Cs2—Sn7—Cs1	68.033 (13)
Sn1^ix^—Cs3—Cs4^viii^	84.558 (15)	Sn6—Sn7—Cs2^vii^	131.71 (2)
Cs4—Cs3—Cs4^viii^	141.674 (15)	Sn4—Sn7—Cs2^vii^	65.197 (16)
Sn5^ix^—Cs3—Cs4^viii^	56.920 (12)	Sn5—Sn7—Cs2^vii^	73.933 (18)
Cs1—Cs3—Cs4^viii^	127.162 (15)	Sn8—Sn7—Cs2^vii^	117.69 (2)
Cs5—Cs3—Cs4^viii^	139.627 (15)	Cs2—Sn7—Cs2^vii^	88.220 (12)
N1—Cs4—Cs5	111.82 (14)	Cs1—Sn7—Cs2^vii^	154.315 (19)
N1—Cs4—Sn1^ix^	107.34 (13)	Sn3—Sn1—Sn4	67.23 (2)
Cs5—Cs4—Sn1^ix^	108.886 (17)	Sn3—Sn1—Sn5	100.91 (2)
N1—Cs4—Sn2^ix^	66.34 (14)	Sn4—Sn1—Sn5	65.72 (2)
Cs5—Cs4—Sn2^ix^	135.927 (17)	Sn3—Sn1—Sn2	66.48 (2)
Sn1^ix^—Cs4—Sn2^ix^	42.824 (13)	Sn4—Sn1—Sn2	101.03 (2)
N1—Cs4—Sn6^v^	165.43 (14)	Sn5—Sn1—Sn2	65.05 (2)
Cs5—Cs4—Sn6^v^	60.155 (14)	Sn3—Sn1—Cs4^xiii^	96.83 (2)
Sn1^ix^—Cs4—Sn6^v^	87.136 (17)	Sn4—Sn1—Cs4^xiii^	163.88 (2)
Sn2^ix^—Cs4—Sn6^v^	128.14 (2)	Sn5—Sn1—Cs4^xiii^	117.52 (3)
N1—Cs4—Cs2	55.63 (14)	Sn2—Sn1—Cs4^xiii^	68.824 (19)
Cs5—Cs4—Cs2	64.497 (14)	Sn3—Sn1—Cs3^xiii^	145.08 (2)
Sn1^ix^—Cs4—Cs2	150.248 (18)	Sn4—Sn1—Cs3^xiii^	130.33 (2)
Sn2^ix^—Cs4—Cs2	120.324 (18)	Sn5—Sn1—Cs3^xiii^	70.26 (2)
Sn6^v^—Cs4—Cs2	110.600 (16)	Sn2—Sn1—Cs3^xiii^	79.59 (2)
N1—Cs4—Cs3	83.55 (13)	Cs4^xiii^—Sn1—Cs3^xiii^	61.826 (14)
Cs5—Cs4—Cs3	67.362 (13)	Sn3—Sn1—Cs2^xi^	97.98 (2)
Sn1^ix^—Cs4—Cs3	61.026 (14)	Sn4—Sn1—Cs2^xi^	66.681 (18)
Sn2^ix^—Cs4—Cs3	68.737 (13)	Sn5—Sn1—Cs2^xi^	115.64 (2)
Sn6^v^—Cs4—Cs3	102.670 (15)	Sn2—Sn1—Cs2^xi^	163.63 (3)
Cs2—Cs4—Cs3	91.084 (16)	Cs4^xiii^—Sn1—Cs2^xi^	120.338 (17)
N1—Cs4—Sn5^vi^	63.77 (13)	Cs3^xiii^—Sn1—Cs2^xi^	116.465 (16)
Cs5—Cs4—Sn5^vi^	114.035 (17)	Sn3—Sn1—Cs1^xi^	61.077 (16)
Sn1^ix^—Cs4—Sn5^vi^	136.305 (18)	Sn4—Sn1—Cs1^xi^	98.27 (2)
Sn2^ix^—Cs4—Sn5^vi^	104.233 (16)	Sn5—Sn1—Cs1^xi^	160.44 (2)
Sn6^v^—Cs4—Sn5^vi^	107.003 (15)	Sn2—Sn1—Cs1^xi^	109.90 (2)
Cs2—Cs4—Sn5^vi^	62.751 (14)	Cs4^xiii^—Sn1—Cs1^xi^	74.730 (14)
Cs3—Cs4—Sn5^vi^	145.75 (2)	Cs3^xiii^—Sn1—Cs1^xi^	128.664 (17)
N1—Cs4—Cs3^x^	69.42 (14)	Cs2^xi^—Sn1—Cs1^xi^	63.520 (13)
Cs5—Cs4—Cs3^x^	167.984 (17)	Cs5—O1—H1	180.0
Sn1^ix^—Cs4—Cs3^x^	81.400 (14)	H4A—N4—H4B	109.5
Sn2^ix^—Cs4—Cs3^x^	55.981 (12)	H4A—N4—H4C	109.5
Sn6^v^—Cs4—Cs3^x^	115.700 (17)	H4B—N4—H4C	109.5
Cs2—Cs4—Cs3^x^	109.695 (15)	H3A—N3—H3B	109.5
Cs3—Cs4—Cs3^x^	124.270 (12)	H3A—N3—H3C	109.5
Sn5^vi^—Cs4—Cs3^x^	55.068 (12)	H3B—N3—H3C	109.5
Sn4—Sn8—Sn3	66.964 (19)	H2A—N2—H2B	109.5
Sn4—Sn8—Sn9	105.62 (2)	H2A—N2—H2C	109.5
Sn3—Sn8—Sn9	59.38 (2)	H2B—N2—H2C	109.5
Sn4—Sn8—Sn7	59.975 (18)	Cs4—N1—H1A	109.5
Sn3—Sn8—Sn7	105.81 (2)	Cs4—N1—H1B	109.5
Sn9—Sn8—Sn7	89.35 (2)	H1A—N1—H1B	109.5
Sn4—Sn8—Cs1	130.47 (2)	Cs4—N1—H1C	109.5
Sn3—Sn8—Cs1	129.70 (2)	H1A—N1—H1C	109.5
Sn9—Sn8—Cs1	70.347 (17)	H1B—N1—H1C	109.5
Sn7—Sn8—Cs1	70.536 (17)		

Symmetry codes: (i) −*x*+3/2, −*y*+1, *z*+1/2; (ii) *x*+1, *y*, *z*; (iii) *x*+1/2, −*y*+3/2, −*z*+1; (iv) −*x*+2, *y*+1/2, −*z*+1/2; (v) −*x*+3/2, −*y*+1, *z*−1/2; (vi) *x*+1/2, −*y*+1/2, −*z*+1; (vii) *x*−1/2, −*y*+1/2, −*z*+1; (viii) −*x*+1, *y*+1/2, −*z*+1/2; (ix) −*x*+1/2, −*y*+1, *z*−1/2; (x) −*x*+1, *y*−1/2, −*z*+1/2; (xi) *x*−1, *y*, *z*; (xii) *x*−1/2, −*y*+3/2, −*z*+1; (xiii) −*x*+1/2, −*y*+1, *z*+1/2.
